# Signal Property Information-Based Target Detection with Dual-Output Neural Network in Complex Environments

**DOI:** 10.3390/s23104956

**Published:** 2023-05-22

**Authors:** Lu Shen, Hongtao Su, Zhi Mao, Xinchen Jing, Congyue Jia

**Affiliations:** National Key Laboratory of Radar Signal Processing, Xidian University, Xi’an 710071, China

**Keywords:** radar signal processing, target detection, signal property information, dual-output network, dynamic-intelligent threshold

## Abstract

The performance of traditional model-based constant false-alarm ratio (CFAR) detection algorithms can suffer in complex environments, particularly in scenarios involving multiple targets (MT) and clutter edges (CE) due to an imprecise estimation of background noise power level. Furthermore, the fixed threshold mechanism that is commonly used in the single-input single-output neural network can result in performance degradation due to changes in the scene. To overcome these challenges and limitations, this paper proposes a novel approach, a single-input dual-output network detector (SIDOND) using data-driven deep neural networks (DNN). One output is used for signal property information (SPI)-based estimation of the detection sufficient statistic, while the other is utilized to establish a dynamic-intelligent threshold mechanism based on the threshold impact factor (TIF), where the TIF is a simplified description of the target and background environment information. Experimental results demonstrate that SIDOND is more robust and performs better than model-based and single-output network detectors. Moreover, the visual explanation technique is employed to explain the working of SIDOND.

## 1. Introduction

Target detection is essential to radar signal processing and plays a vital role in all sensor fields. For radar systems, it means deciding whether radar data represent an echo coming from a target. The presence of a target prompts the system to engage in further processing [1]. However, the robustness of the detection algorithms may suffer due to the complexity and dynamic variability of the environment, which can be broadly categorized into three scenarios [2]. The first is homogeneous background. In this model, the stationary background noise exists throughout the reference window. The second is the clutter edge model. This model describes the transition areas between different background regions. The third scenario is multiple targets. This situation represents two or more spatially close targets in the detection window.

Radar target detection can be achieved by either model-based or data-driven detectors, where the former employs statistical models to build a likelihood-ratio test (LRT), while the latter transforms the task of target detection into a classification problem. According to the Neyman–Pearson criterion, the model-based constant false-alarm ratio (CFAR) technique can maintain a constant probability of false alarms ($P_{fa}$) while maximizing the probability of detection ($P_{d}$), which provides an adaptive detection threshold for the LRT by estimating the cell-under-test (CUT) background noise power level (BNPL) using reference cells adjacent to the CUT. According to the BNPL estimation strategy, CFAR algorithms can be divided into three categories, mean-level (ML), ordered statistics (OS), and adaptive CFAR. The ML CFAR algorithms, such as cell average CFAR (CA-CFAR) [3], the smallest-of CFAR (SO-CFAR) [4], and the greatest-of CFAR (GO-CFAR) [5], estimate BNPL by weighted averaging of leading, lagging, or the entire reference window samples. They can provide accurate BNPL estimates in a homogeneous, independent, and identically distributed (iid) environment. The OS CFAR (e.g., ordered statistical CFAR (OS-CFAR) [2], the trimmed mean CFAR [6], and the censored mean-level detector [7]) estimate BNPL from an ordered sequence of samples within the reference window, providing better performance in multiple target environments. The adaptive CFAR can adaptively determine the logic, algorithms, and parameters for estimating BNPL. The adaptive censored greatest-of CFAR [8] adaptively determines deletion points based on ordered statistics and removes interfering targets one by one to obtain high detection performance in multiple targets scenarios. The variable index CFAR (VI-CFAR) [9] adaptively determines the detection algorithm based on the uniform statistics characteristics of background clutter. The robust variable index CFAR [10] determines an adaptive threshold in the first stage and rejects outlier in subsequent stages. The robust variability index CFAR, based on Bayesian interference control theory (BVI-CFAR) [11], adaptively evaluates the BNPL by uniformly partitioning the clutter region and optimizing the selection strategy. However, model-driven target detection algorithms are susceptible to model mismatch, resulting in sensitivity to changes in the statistical model of the underlying data. Moreover, the presence of interfering targets and clutter edges can introduce non-homogeneities in the reference window samples, leading to a reduction in performance for the aforementioned algorithms.

For data-driven detectors, detecting intrinsic features and constructing efficient classifiers are essential for improving the performance of data-driven detectors. Previous research has focused on utilizing data-driven machine learning to address detection problems [12,13,14]. For instance, the study by Zhai et al. [15] proposes a reinforcement-based target detection and communication system for massive multiple-input multiple-output arrays that effectively enhances the multi-target scenario target detection capability. While power allocation for antenna transmission is a prevalent algorithmic approach, this paper primarily focuses on the signal processing stage after receiving the echo. Coluccia et al. proposed a radar detector based on the k-nearest neighbor (KNN) approach [16], while Wang et al. developed a detector based on residual networks to detect high-speed targets with phase-encoded signals [17]. In addition, Gao et al. used a signal structure information-based convolutional neural network (CNN) for target detection [18]. These algorithms are typically considered as single-input single-output network detectors (SISONDs). SISONDs usually set a constant threshold, which is determined by the worst-case scenario, to achieve the desired $P_{fa}$. However, the fixed threshold can be mismatched and result in degradation in performance due to dynamic and time-varying complex environments. In complex backgrounds, increasing the capacity of the network architecture and the number of training samples may improve detection performance. However, high-capacity networks may extract more abstract features, which would come at the cost of computational, memory, and training complexity, making them challenging to train for complex target detection problems compared to simpler scenes. Therefore, SISONDs may only be effective in specific environments, and changes in the scene can result in performance degradation due to hard-training networks and threshold mismatches.

In this paper, a single-input dual-output network detector (SIDOND) is proposed to alleviate the limitations of the fixed threshold approaches used in SISOND. The objective is to achieve optimal detection performance and robustness in complex environments. The proposed SIDOND employs two sub-networks to exploit the intrinsic information of the reflected signals for detecting targets. One sub-network is responsible for estimating detection sufficient statistics (DSS) for feature-based classification, while the other sub-network is dedicated to estimating the threshold impact factor (TIF) which forms the basis of a dynamic-intelligence threshold mechanism. In radar applications, target echo typically contains significant intrinsic structure information related to the transmitted waveform and the target itself. A data-driven method that recognizes and leverages this intrinsic structure information would successfully solve the detection task. Despite the potential benefits of signal properties information (SPI) for target detection, to the best of our knowledge, its impact on detection performance has not been adequately studied in the open literature. Furthermore, the proposed method exploits all the available information from the detection window, which includes target echoes, interfering echoes, clutter, and other relevant data to determine the optimal threshold for the SPI-based detector. The TIF is a compact representation of the significant information in the detection window. A higher TIF value reflects the presence of more discernible SPI in the detection window, leading to a lower threshold requirement for the detector. By employing a dynamic-intelligent threshold mechanism, the proposed SIDOND can effectively enhance the target detection performance in complex environments.

The main contributions of this paper are as follows:

1. The proposed single-input double-output network detector (SIDOND) is a promising approach to extracting both the target and background environment features without significantly increasing network capacity and training complexity.
2. The dynamic-intelligent threshold mechanism can adaptively adjust the threshold based on the estimated target and environmental information, which enhances the detection performance in a complex environment while maintaining a low false-alarm rate.
3. The CNN based on periodic activation function and a particular initialization strategy can effectively avoid the gradient disappearance problem of deep networks, which improves the convergence speed and network performance in the target detection task.

The remaining sections of this paper are organized as follows. In Section 2, the model of target echos is introduced, and the target detection task is formulated. Section 3 analyzes the methodology and structure of the proposed SIDOND. Section 4 presents simulation results under various conditions, including multiple targets, clutter edges, and complex environments. Finally, Section 5 provides conclusions.

Some symbols used in this paper are explained as follows. The boldface characters represent vectors or matrices. $N\left(\mu ,\sigma^{2}\right)$ is defined as a normal distribution with mean μ and variance $\sigma^{2}$. ⊙ stands for Hadamard product. ⊗ stands for convolution operation. ${\left(\cdot \right)}^{T}$ represents the transpose.

## 2. Problem Formulation

### 2.1. Signal Model

The echo signal of a target in a radar system can be approximately modeled as [1]

(1) $$x\left(t\right)=kA\left(t-\frac{2R_{0}}{c}\right)\exp\left(-j\frac{4\pi }{\lambda }\left(R_{0}+vt\right)\right)+n\left(t\right),$$

where *k* contains all of the factors related to amplitude in the radar range equation. $R_{0}$ is the distance from the target to the radar, and A(t) is the baseband transmit waveform. *v* is the target radial velocity, *c* represents the propagation speed of electromagnetic waves, and λ is the wavelength. n(t) here is clutter and noise.

A(t), the signal waveform, is the critical information feature for target detection. Without loss of generality, the linear frequency modulated (LFM) signal is used as the transmit signal waveform, which can be stated as

(2) $$A\left(t\right)=\exp\left(j\pi t^{2}\beta /\tau \right),0\leq t\leq \tau ,$$

where β is bandwidth and τ is pulse width. The received signal is sampled with the frequency $F_{s}$ and the corresponding sampling interval is $T_{s}=1/F_{s}$. In the received data x, the target will affect the *q*-th to the (q+L)-th samples, where $q=2R_{0}F_{s}/c$, $L=\tau F_{s}$.

Then, the echo can be stated as $[x_{q},x_{q+1},x_{q+2},\cdots ,x_{q+L-1}]$, where

(3) $$\begin{array}{c} x_{q+l}=kA_{l}\exp\left(-j4\pi R_{0}/\lambda \right)\cdot \exp\left(f_{v}\left(q+l\right)\right)+n\left(q+l\right),0\leq l\leq L-1, \end{array}$$

where $A_{l}=\exp [j\pi \beta {\left(T_{s}l-2R_{0}/c\right)}^{2}/\tau ]$, $f_{v}=j2\pi \left(2v/\lambda \right)T_{s}$. Then, the radar echo data segment $x_{q}^{L}= [x_{q},x_{q+1},x_{q+2},\cdots ,x_{q+L-1}]$ related to the target can be simplified as

(4) $$x_{q}^{l}=\tilde{k}A⊙F_{v}+n,$$

where $\tilde{k}=k\exp\left(-j4\pi R_{0}/\lambda \right)$, $A= [A_{0},A_{1},A_{2},\cdots ,A_{l-1}]$ and $F_{v}$ is the Doppler modulation caused by target radial velocity $F_{v}=[\exp\left(f_{v}q\right),\cdots ,$ $\exp\left(f_{v}\left(q+L-1\right)\right)]$ [19].

**Figure 1:**
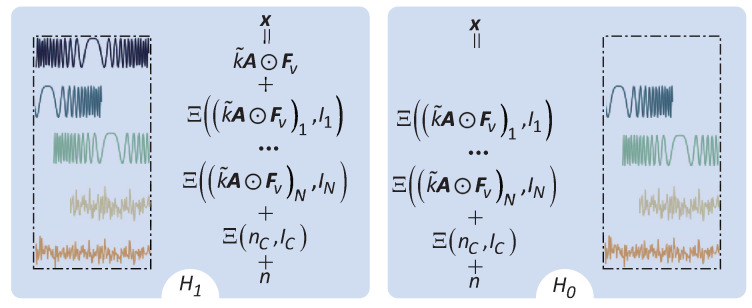
The sensor echo model.

It needs to be stated that the detection window length should be set as the waveform length *L* in order to acquire the complete information of the transmit waveform. In complex environments, each detection window contains not only target echo but also interference echo, clutter edges, and noise. Based on the detection principle, two hypotheses are defined for the target echo: the null hypothesis $H_{0}$ and the non-null hypothesis $H_{1}$ [1]. The $H_{1}$ means that the detection window contains a complete transmit waveform. Figure 1 shows a schematic diagram of these hypotheses.

In Figure 1, the translation symbol Ξ(s,n) represents shift of the vector s to the left (n<0) or right (n>0) by *n* sampling points. Then, the detection problem can be written as

(5) $$\left\{\begin{array}{c} H_{0}:x=\sum_{r=1}^{N_{I}}\Xi ({\left(\tilde{k}A⊙F_{v}\right)}_{r},I_{r})+\Xi \left(n_{C},I_{C}\right)+n\\ H_{1}:x=\tilde{k}A⊙F_{v}+\sum_{r=1}^{N_{I}}\Xi ({\left(\tilde{k}A⊙F_{v}\right)}_{r},I_{r})+\Xi \left(n_{C},I_{C}\right)+n, \end{array}\right.$$

where $N_{I}$ is the number of interference, $n_{C}$ represents the sudden change of clutter power, *I* and $I_{C}$ are shift samples at the edge of interference and clutter, and $-L\leq I,I_{C}\leq L-1$. It can be seen that the SPI exists in the detection window.

### 2.2. Posterior Probability Detector

According to the Bayesian detection criteria, the problem in (5) can be solved by constructing a likelihood ratio detector,

(6) $$\Lambda \left(x\right)=\frac{f(x|H_{1})}{f(x|H_{0})}\overset{H_{1}}{\underset{H_{0}}{≷}}\frac{P(H_{0})}{P(H_{1})}\cdot \eta ,$$

where $f(x|H_{1})$ and $f(x|H_{0})$ are the probability density of x under $H_{1}$ and $H_{0}$, respectively, $\Lambda \left(x\right)$ is the likelihood ratio, $P(H_{0})$ and $P(H_{1})$ represent the prior probabilities of $H_{1}$ and $H_{0}$. According to Bayes’ theorem, (6) can be recast as

(7) $$P(H_{1}|x)\overset{H_{1}}{\underset{H_{0}}{≷}}P(H_{0}|x)\cdot \eta ,$$

where $P(H_{1}|x)$ and $P(H_{0}|x)$ are the posterior probabilities of $H_{1}$ and $H_{0}$, $P\left(H_{1}|x\right)+P\left(H_{0}|x\right)=1$. The decision rule (7) can be rewritten as

(8) $$\Lambda^{\prime }\left(x\right)=P(H_{1}|x)\overset{H_{1}}{\underset{H_{0}}{≷}}\frac{\eta }{1+\eta }=\eta^{\prime },$$

where the posterior probability is the sufficient statistics and the $\Lambda^{\prime }\left(\cdot \right)$ is the map between the posterior probability and x. Theoretically, the detection threshold $\eta^{\prime }$ can be found from

(9) $$P_{fa}=\int_{\left\{x:P\left(H_{1}|x\right)>\eta^{\prime }\right\}}f\left(x|H_{0}\right).$$

In radar systems, the estimation of detection threshold $\eta^{\prime }$ is frequently accomplished using samples in reference cells, which are independent and identically distributed from the noise in the CUT. Nevertheless, in complex environments, it is crucial to have a threshold that is dynamic and adaptive to the changing conditions. The appropriate threshold selection involves a trade-off between maintaining a constant false-alarm rate and maximizing the probability of target detection. To address these challenges, a dual-output network structure is utilized to dynamically adjust the threshold in complex environments which provides an effective way to estimate the threshold and enhance target detection performance.

## 3. Target Detection Using the SIDOND

**Figure 2:**
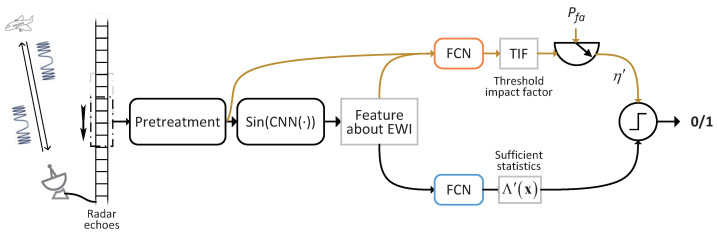
Architecture and flow graph of the SIDOND.

The proposed SIDOND is presented in Figure 2, which depicts its architecture and flow graph. The raw data are first pre-processed and then input into PBCN, which is the CNN based on the periodic activation function (PAF), to extract the intrinsic features of the SPI. The TIF is then obtained through a TIF estimator based on a fully connected network (FCN), called TIFEFCN, which takes both the combination of features and pre-processed data as its input. Additionally, the SPI feature is fed to the detection sufficient statistic estimator based on FCN (DSSEFCN). Finally, the estimated sufficient statistic is compared with the threshold $\eta^{\prime }$, determined based on the TIF and predefined $P_{fa}$, to achieve the detection task. It should be noted that unlike conventional classification algorithms aimed solely at achieving high accuracy, the proposed algorithm focuses on maximizing the detection probability while maintaining an approximate constant $P_{fa}$.

### 3.1. The PBCN for SIDOND

The primary component of PBCN is the CNN, which has demonstrated remarkable performance in various fields, including computer vision [20,21], medical diagnosis [22], target recognition [23,24,25], and signal detection. This study selects CNN as the feature extractor for SPI, with the PAF being used as the an activation function. The use of a non-linear activation function is a fundamental aspect of neural network architectures as it allows for the network to model complex non-linear relationships between inputs and outputs. In particular, non-linear activation functions enable neural networks to achieve excellent fit capabilities. Furthermore, this paper proposed a new CNN initialization scheme based on the PAF, which maintains the distribution of output and input of different layers to achieve faster and better convergence while avoiding undesirable situations, such as gradient vanishing in a deep CNN.

**Figure 3:**
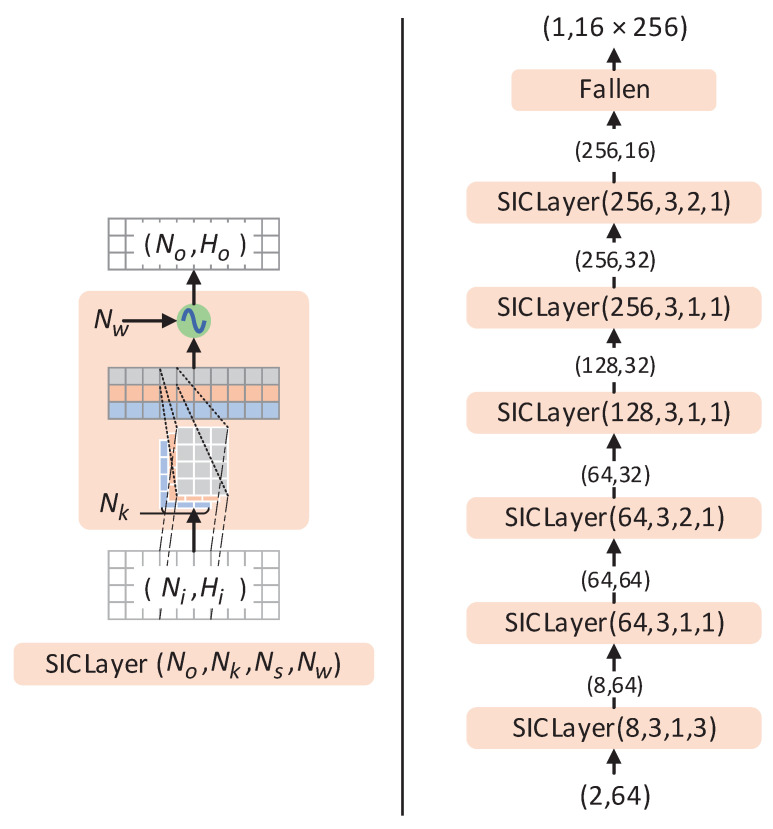
The structure of the SICLayer (left) and the flowchart of PBCN (right). The parameters $\left(N_{o},N_{k},N_{s},N_{w}\right)$ are (the number of output elements, the size of the kernel, the convolution step length, and the expansion factor of PAF).

The convolutional layer that employs the PAF is called SICLayer, and its design and operation are illustrated in Figure 3. The layer has four parameters: $N_{o}$, which is the dimension of the output data; $N_{k}$, which represents the size of the convolution kernel; $N_{s}$, which is the convolution step length; and $N_{w}$, which is the expansion factor that can be adjusted to a higher value in the first few layers to preserve more comprehensive feature information.

The input of the *l*-th SICLayer is $Z^{l}$ with a dimension of $N_{i}\times H_{i}$, the output is ${\hat{Z}}^{l}$ of dimension $N_{o}\times H_{o}$, and the size of the convolution kernel is $1\times N_{k}^{l}$, where the required convolution parameter is the weight $w^{l}$ with dimension $N_{o}\times N_{k}^{l}\times N_{i}$ and the bias $b^{l}$ with dimension $N_{o}\times 1$. Then the output of convolutional is

(10) $${\hat{Z}}_{n,:}^{l}=\sum_{j=1}^{N_{i}}Z_{j,:}^{l}⊗w_{n,:,j}^{l}+b_{n}^{l},n=1,2,\ldots ,N_{o}.$$

The subscripts specify the position of the element in the raw data. For example, ${\hat{Z}}_{n,:}^{l}$ represents all elements of ${\hat{Z}}^{l}$ whose first dimension is *n*. Afterwards, the output of SICLayer is

(11) $$Z^{l+1}=\sin\left(N_{w}^{l}{\hat{Z}}^{l}\right).$$

The performance of a network is significantly affected by its initialization [26]. The convolution kernel is represented by a weight matrix w with three dimensions: the input depth $H_{i}$, the kernel length $N_{k}$, and the output depth $H_{o}$.

The initialization of w obeys the uniform distribution of [−c,c], that is $w∼U\left(-c,c\right)$, where *c* is

(12) $$c=\left\{\begin{array}{c} \sqrt{6/\left(N_{k}H_{i}\right)}/N_{w},\mathrm{Non}-\mathrm{firstlayer}\\ \sqrt{3/\left(N_{k}H_{i}\right)}/N_{w},\mathrm{Firstlayer}. \end{array}\right.$$

In other words, the *c* is related to the depth dimension $L^{i}$ of the input data, kernel length $N_{k}$, and the expansion factor $N_{w}$ of the periodic activation function. With such an initialization, the data before the periodic activation function approximately obeys the standard normal distribution, and the data after the PAF approximately obeys the arcsine distribution. The proof is presented in Appendix A.

### 3.2. The Structure of the DSSEFCN

**Figure 4:**
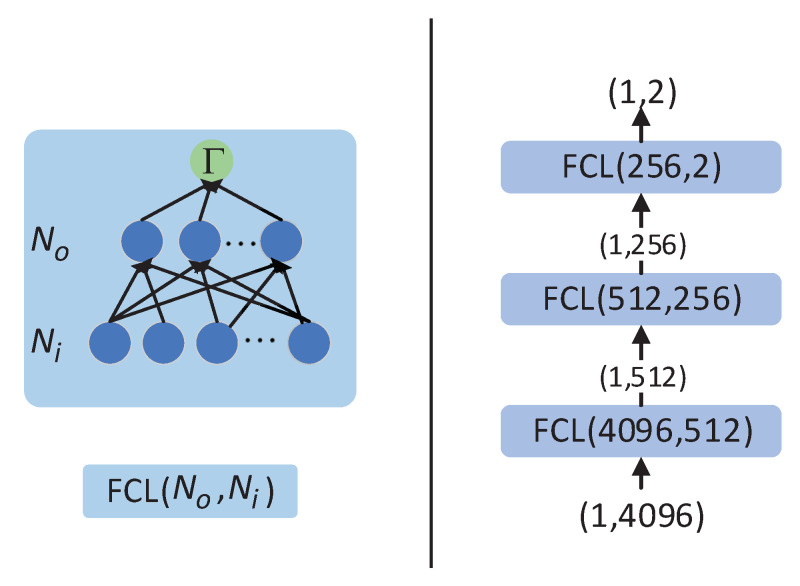
Fully connected network structure (**left**) and the flowchart of DSSEFCN (**right**). The parameters $\left(N_{i},N_{o}\right)$ are (the number of input elements, the number of output elements).

The FCN is the fundamental unit for DSSEFCN, and it has been widely applied in the field of neural network development due to its efficacy in addressing classification and regression problems. Accordingly, the FCN is employed to construct the posterior probability estimator in this paper. The configuration of the fully connected layer (FCL) and the DSSEFCN is presented in Figure 4.

Assuming that the input of the *l*-th FCN is $Z^{l}$ whose dimension is $1\times N_{i}$, the output is $Z^{l+1}$ with the dimension $1\times N_{o}$, and the weight vector $w^{l}$ with required dimension $N_{i}\times N_{o}$ and the bias $b^{l}$ of dimension $1\times N_{o}$. The activation function in the figure is Γ. Thus, the output is

(13) $$Z^{l+1}=\Gamma \left(Z^{l}w^{l}+b^{l}\right).$$

The activation function commonly used in hidden layers of FCN is rectified linear unit (ReLU) [27]. To retain more information and features, the LeakyReLU function [28] is specially used, and its expression is

(14) $$LeakyRelu\left(x\right)=\max\left(0,x\right)+0.01\times \min\left(0,x\right).$$

The SoftMax [29] is used as the output layer activation function. When the input is x of length *n*, the *j*-th SoftMax output is

(15) $$SoftMax\left(x_{j}\right)=e^{x_{j}}/\sum_{k=1}^{n}e^{x_{k}}.$$

The output of the DSSEFCN is the approximation of sufficient statistics and the associated expression can be defined as follows:

(16) $$\Lambda_{net}^{\prime }\left(x\right)=P_{net}\left(H_{1}|x\right)=DSSEFCN\left(PBCN\left(x\right)\right),$$

where the $P_{net}\left(H_{1}|x\right)$ is the posterior probability approximated by the network. The $DSSEFCN\left(\cdot \right)$ and the $PBCN\left(\cdot \right)$ are cascades of multi-layer FCL and multi-layer SICLayer, respectively.

### 3.3. Dynamic-Intelligent Threshold Mechanism

After obtaining the estimate of the posterior probability $P_{net}\left(H_{0}|x\right)$, selecting the threshold $\eta^{\prime }$ is crucial for solving the target detection problem. It can be seen from (9) that $\eta^{\prime }$ is determined by $P_{fa}$, $P\left(H_{1}|x\right)$ and $f\left(x|H_{0}\right)$. Under the background of Gaussian noise, the probability density of x obeys the N-dimensional independent joint Gaussian distribution, which can be formulated as

(17) $$f\left(x|H_{0}\right)=\prod_{i=1}^{N}\frac{1}{\sqrt{2\pi }\sigma_{i}}exp\left(-\frac{{\left(x_{i}-\mu_{i}\right)}^{2}}{2\sigma_{i}^{2}}\right),$$

where the noise power and clutter edge determine $\sigma_{i}$. When the transmit waveform is fixed, the $\mu_{i}$ is determined by the power of the interfering target signal. Substituting $\mu = [\mu_{1},\mu_{2},\ldots ,\mu_{N}]$ and $\sigma = [\sigma_{1},\sigma_{2},\ldots ,\sigma_{N}]$, the map on threshold $\eta^{\prime }$ can be formulated as

(18) $$\eta^{\prime }\leftarrow \left\{P_{net}\left(H_{1}|x\right),P_{fa},\mu ,\sigma \right\}.$$

From (18), the threshold $\eta^{\prime }$ is dynamic since the variable parameters are {σ,μ}, and its exact mathematical expression is extremely hard to derive because of the inability to accurately estimate $\left\{P\left(H_{1}|x\right),\sigma ,\mu \right\}$ under complex changing scenarios.

**Figure 5:**
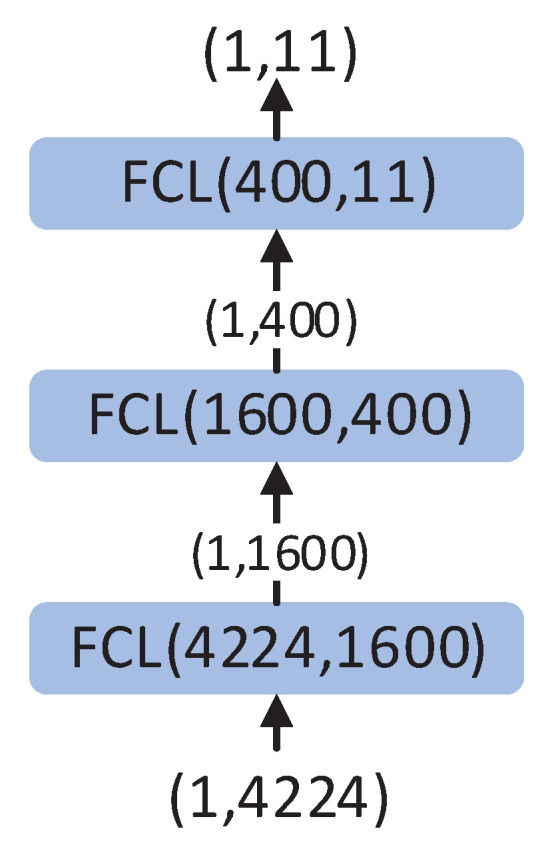
The structure of the neural network part of the TIF estimation.

To address this, using TIF to characterize {σ,μ}, a mechanism that utilizes TIF to approach the optimal threshold is proposed, which can effectively enhance $P_{d}$ and ensure the $P_{fa}$ requirements. Specifically, an TIFEFCN is employed to estimate TIF, as shown in Figure 5. The main building blocks of TIFEFCN are the same as those of the DSSEFCN, which has been discussed in Section 3.2. The extracted features related to the posterior probability and the original data x are fed into this TIFEFCN. The TIF is categorized into a predetermined number of labels that correspond to different signal to noise ratio (SNR) or interference-to-noise ratio (INR) intervals. By jointly estimating TIF and the preset $P_{fa}$, the current threshold is determined.

Thus far, the fundamental building blocks of the SIDOND architecture have been proposed, which include the PBCN, DSSEFCN, and TIFEFCN. In order to assess the performance of the proposed SIDOND, a single-input single-output detector (SISOND) utilizing a PBCN and DSSEFCN is built for comparison purposes.

## 4. Simulations

### 4.1. Simulation Setup

#### 4.1.1. Experimental Data

All experiments in this paper are based on simulation, and all signals without special instructions are generated by the signal model given in Section 2. The sensor receiving the signal is set as an LFM signal, and the specific parameters are shown in Table 1. The pre-treatment in Figure 2 includes normalization and splitting the complex values into real and imaginary parts.

In the experiments of this paper, the computation of $P_{fa}$ is not direct. Hence, Monte Carlo strategy is used to estimate $P_{fa}$. Assuming that the Monte Carlo estimation of the false-alarm rate is ${\hat{P}}_{fa}$, it approximately obeys a Gaussian distribution according to the central limit theorem.

(19) $${\hat{P}}_{fa}∼N\left(P_{fa},\frac{P_{fa}\left(1-P_{fa}\right)}{K}\right),$$

where *K* is the number of Monte Carlo trials. Then the false alarm rate error can be calculated as

(20) $$e=\left({\hat{P}}_{fa}-P_{fa}\right)∼N\left(0,\frac{P_{fa}\left(1-P_{fa}\right)}{K}\right).$$

Setting a tolerance error as *E*, the probability of meeting the tolerance requirement is

(21) $$P\left(\left|e\right|<E\right)=1-2Q\left(\frac{E}{\sqrt{P_{fa}\left(1-P_{fa}\right)/K}}\right),$$

where *Q* is the complementary Gaussian cumulative distribution function. Then, the condition that satisfies the tolerance with a certain probability can be obtained by

(22) $$K\geq { [Q^{-1}\left(\frac{1-P\{\left|e\right|<E\}}{2}\right)]}^{2}\frac{P_{fa}\left(1-P_{fa}\right)}{E^{2}}.$$

For example, setting $P_{fa}$ to be 0.0001, *E* to be 0.000025, and $P(\left|e\right|<E)$ to be 90%, the number of Monte Carlo experiments is at least 432,843.

Training data set: A total of $1\times 10^{7}$ data were generated In this paper, with an $H_{1}:H_{0}$ ratio of 1:1. To facilitate the feature extraction based on the SPI, the interfering target number $N_{I}$ was set to 1, and the clutter edge $n_{C}$ was set to 0 in the PBCN. The SNR or INR followed a uniform distribution on the integer set $[-13,5]$ dB. When only noise was present, the noise power $\sigma^{2}$ followed a uniform distribution on the integer set $[-5,13]$ dB.

Test data set: The details of the test data set will be explained in each respective test section.

#### 4.1.2. The Process of Training the Network

First, the PBCN and DSSEFCN are trained. The binary classification task corresponds to the binary label y∈{0,1}. When y=0, it represents $H_{0}$, and when y=1, it means $H_{1}$. Given x, label *y* obeys Bernoulli distribution

(23) $$p\left(y|x\right)=P{\left(H_{1}|x\right)}^{y}P{\left(H_{0}|x\right)}^{1-y}.$$

For the labels to be considered and the output of the neural network, there are

(24) $$p_{net}\left(y|x\right)=P_{net}{\left(H_{1}|x\right)}^{y}P_{net}{\left(H_{0}|x\right)}^{1-y},$$

where $P_{net}\left(H_{1}|x\right)+P_{net}\left(H_{0}|x\right)=1$.

Relative entropy, also known as Kullback–Leibler (KL) divergence or information divergence, is a type of statistical distance. The relative entropy can measure the difference between the information entropy of the actual distribution $P\left(H_{1}|x\right)$ and the cross-entropy of $P\left(H_{1}|x\right)$ and $P_{net}\left(H_{1}|x\right)$, representing the information loss caused by the fitting distribution. The relative entropy is

(25) $$D_{KL}\left(p\left(y|x\right)|\right|p_{net}\left(y|x\right))=E_{x∼P(y|x)}\left[\log p\left(y|x\right)-\log p_{net}\left(y|x\right)\right].$$

In (25), only $p_{net}\left(y|x\right)$ can be optimized by our algorithm, the loss function is formulated as

(26) $$Loss=E_{x∼p(y|x)}\left[-\log p_{net}\left(y|x\right)\right].$$

When the parameters of PAF-based CNN and FCN are defined as W, the process of obtaining the best W can be regarded as an optimization problem

(27) $$\begin{array}{cc} W & =\underset{W}{\arg\min}Loss=\underset{W}{\arg\min}E_{x∼p(y|x)}\left[-\log p_{net}\left(y|x\right)\right]\\ & =\underset{W}{\arg\min}-\left(y\log\left[P_{net}\left(H_{1}|x\right)\right]+\left(1-y\right)\log\left[P_{net}\left(H_{0}|x\right)\right]\right). \end{array}$$

In other words, the maximum-likelihood method can be used to train the network. However, it is not feasible to obtain the optimal global solution for W since (26) is highly non-convex. Nevertheless, effective gradient descent optimization methods can yield acceptable solutions. Given a training batch consisting of *N* output data $[z^{1},z^{2},z^{3},\ldots ,z^{N}]$ and labels $[y^{1},y^{2},y^{3},\ldots ,y^{N}]$, then the cost function is

(28) $$J=\sum_{i=1}^{N}y^{i}\ln\left(z^{i}\right)+\left(1-y^{i}\right)\ln\left(1-z^{i}\right).$$

The back-propagation algorithm is employed to train the neural network, and the SGD algorithm with an initial learning rate of 0.001 and momentum of 0.99 is used for optimization [30]. The learning rate is reduced by four-fifths every ten epochs. The experiment is conducted on TensorFlow-GPU 2.0.

Subsequently, the Monte Carlo integration method can be utilized to derive the threshold based on a preset value of $P_{fa}$.

(29) $$P_{fa}=\frac{1}{N}\sum_{n=1}^{N}I [P_{net}\left(H_{1}|x^{n}\right)>\eta^{\prime },x^{n}∼f\left(x^{n}|H_{0},\mu ,\delta \right)].$$

**Figure 6:**
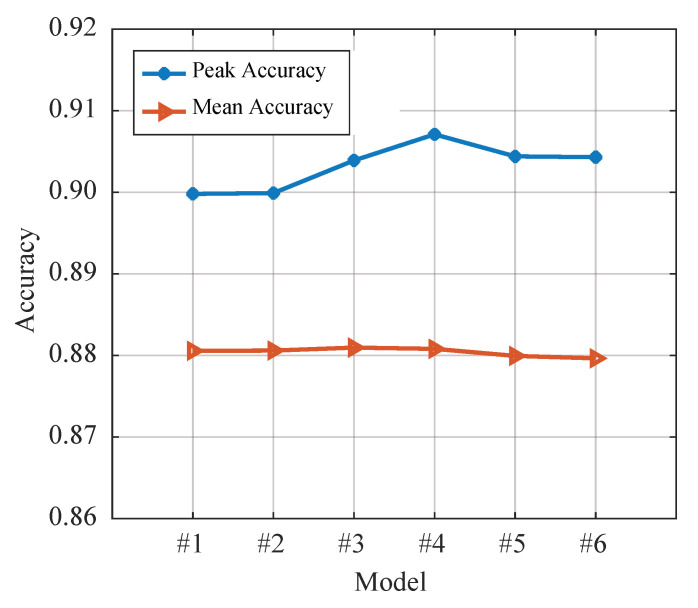
Loss convergence under different activation functions.

The $P_{d}$ can be formulated by

(30) $$P_{d}=\frac{1}{N}\sum_{n=1}^{N}I [P_{net}\left(H_{1}|x^{n}\right)>\eta^{\prime },x^{n}∼f\left(x^{n}|H_{1},\mu ,\delta \right)],$$

where $I\left[\cdot \right]$ denotes the indicator function. To compare the impact of different network architectures on performance, six models with varying numbers of layers and nodes have been generated, and their specific parameters are presented in Table 2. Each model is trained for four epochs on the training set, and a test dataset is generated with the same parameters as the training set to evaluate the algorithm’s performance. The average and peak accuracy of each model are shown in Figure 6. Notably, the performance of PBCN is relatively consistent across different parameter settings, indicating that it is not highly sensitive to network architecture. Additionally, it can be seen that when the number of layers exceeds six, the algorithm’s performance improves gradually or even deteriorates. Based on these results, this paper has selected the #4 network for subsequent experiments. However, in practical applications, the network size can be adjusted according to hardware and other requirements.

**Figure 7:**
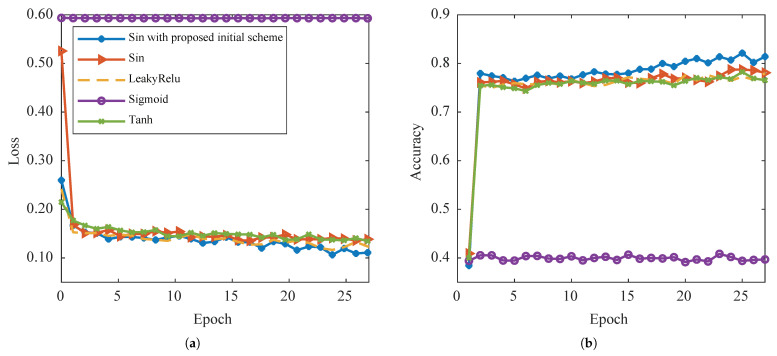
Loss convergence (**a**) and accuracy on the test set (**b**) under different activation functions. The test set is composed of target and multiple interferences.

The performance comparison of different activation functions and initialization methods was conducted in this paper, and the results are presented in Figure 7. The PAF-activated network with the proposed novel initialization scheme is observed to converge well, as depicted in Figure 7. The accuracy performance of the network is evaluated on two related test sets during the training process, and it is observed that the proposed PAF and initialization method outperforms other methods for both test sets. The results suggest that the proposed method is effective in improving the performance of the network.

A new training dataset is generated by restricting the values of SNR and INR parameters within the range of [−19, 5] dB. The corresponding TIF values are assigned based on Table 3. These TIF values are used as the training labels for FCN. The network is trained with the same training parameters and loss function for 30 epochs to achieve the required accuracy. Multiple sets of data are used to conduct Monte Carlo experiments, from which a mapping table is obtained that shows the relationship among TIF, threshold, and $P_{fa}$.

As waveform information is a key feature in this study, the algorithm’s performance is evaluated when the parameters of waveform are changed. This comparison focuses on the bandwidth, sampling frequency, pulse width, and number of pulse sampling points. The model was trained with an equal number of samples, and validation was conducted after 30 epochs. Results are presented in Table 4, which indicates that changes in signal bandwidth and sampling frequency do not have a significant effect on the algorithm’s performance. However, an increase in pulse width and the corresponding increase in sampling points improves the algorithm. This result demonstrates the effectiveness of our proposed algorithm in addressing the challenges of target detection in the presence of varying waveform parameters.

### 4.2. Performance Results

This subsection compares the performance of the proposed SIDOND algorithm with several traditional CFAR-based methods, including CA-CFAR [3], SO-CFAR [4], GO-CFAR [5], OS-CFAR [2], VI-CFAR [9], BVI-CFAR [11], as well as a data-driven method called SISOND. The traditional algorithms use 20 reference cells and 6 guard cells, except for BVI-CFAR, which uses 32 reference cells. CA-CFAR, SO-CFAR, and GO-CFAR are different mean-level CFAR methods, while OS-CFAR is an ordered statistical CFAR that uses the 15th-ordered statistic for background noise estimation. VI-CFAR is an adaptive CFAR method that uses two statistics, the variability index (VI) and the mean ratio (MR), set as 4.76 and 1.806. BVI-CFAR is an adaptive CFAR method based on VI and Bayesian interference control theory, with VI and MR set to 5 and 3. The number of interfering targets and clutter range partition is set to 4 and 16, which is a commonly used configuration in the literature. In addition to the traditional CFAR-based methods, the data-driven method SISOND is also considered, which is a single-input single-output echo waveform-based method. The desired $P_{fa}$ is set to 0.0001.

To evaluate the performance of the methods, three different scenes are considered: homogeneous background, multiple targets, and complex environment.

#### 4.2.1. Homogeneous Background

**Figure 8:**
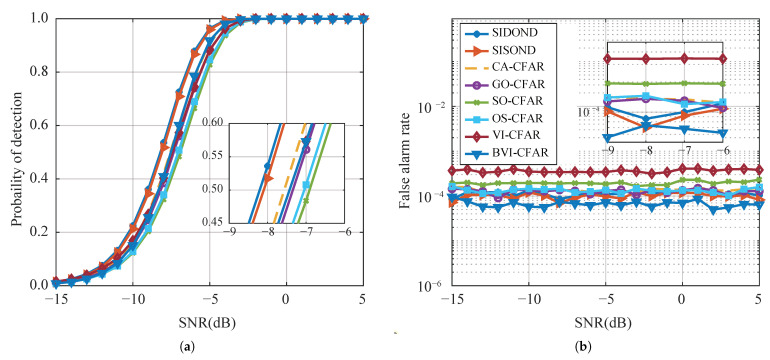
Detection performance (**a**) and false alarm rate (**b**) with a single target in homogeneous environments.

The homogeneous background scene is a common scenario in radar applications, and it serves as a benchmark to evaluate the performance of different detection algorithms. To ensure the accuracy of the Monte Carlo experiment, a set of data was generated in a Gaussian white noise environment, with at least $1\times 10^{6}$ data points at each SNR. Figure 8 presents the results of the experiment in the single-target scenario. Traditional methods such as CA-CFAR, SO-CFAR, GO-CFAR, OS-CFAR, and VI-CFAR exhibit good detection performance, with a maintained $P_{fa}$ of around 0.0001. However, the proposed SIDOND achieves the best detection performance among all methods, thanks to its intelligent threshold mechanism. It shows a slight advantage over the data-driven SISOND, indicating the effectiveness of the proposed method.

#### 4.2.2. Multiple Targets Situation

A test dataset was generated that includes one or multiple interfering targets with amplitudes ranging from 1.0 to 1.2 times that of the target under test. The interfering targets were randomly and uniformly distributed in the reference cells.

**Figure 9:**
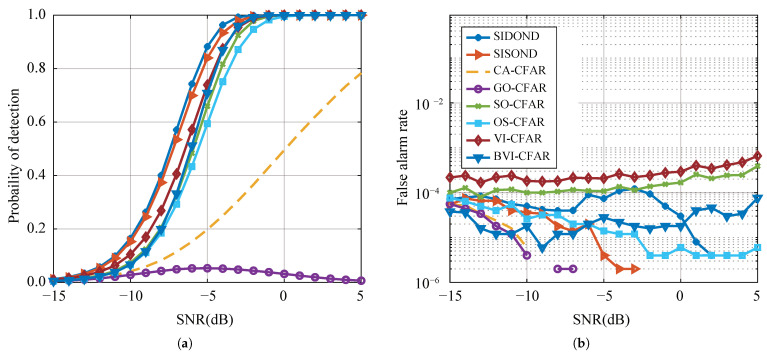
Detection performance (**a**) and false-alarm rate (**b**) with one interference.

**Figure 10:**
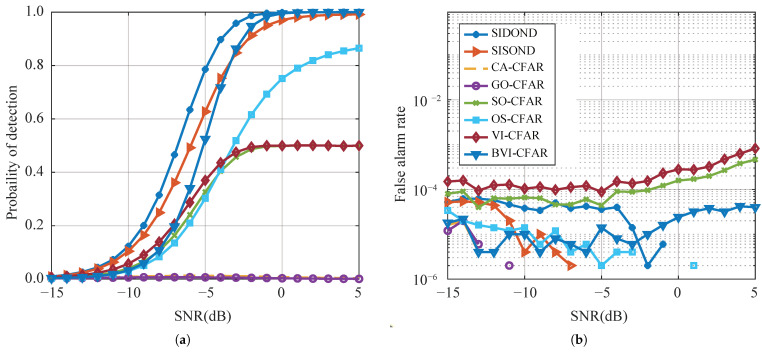
Detection performance (**a**) and false-alarm rate (**b**) with two interferences.

Figure 9 and Figure 10 show that, with the exception of SIDOND, the performance of other detection algorithms deteriorates significantly when multiple interfering targets are present in the reference cell. This degradation is mainly due to the randomly distributed interfering targets in the leading and lagging windows. BVI-CFAR is the most robust of all traditional methods as it can modify the reference cell model based on the uniformity of the reference cell, thereby changing the Bayesian statistics. Under this strategy, BVI-CFAR can maintain a better performance. OS-CFAR estimates the noise power by sorting the power of the reference cells, which effectively avoids the influence of interference. CA-CFAR and GO-CFAR have the worst performance among the traditional methods. However, SIDOND can identify the interfering targets in the detection window and effectively reduce performance loss by obtaining an intelligent threshold. The SIDOND has better $P_{d}$ and $P_{fa}$ maintenance than SISOND, and this advantage increases with the number of interference targets. The discontinuity observed in Figure 9b and Figure 10b for the $P_{fa}$ curves is attributed to the Monte Carlo simulation process where $P_{fa}$ becomes zero and cannot be represented in exponential coordinates.

**Figure 11:**
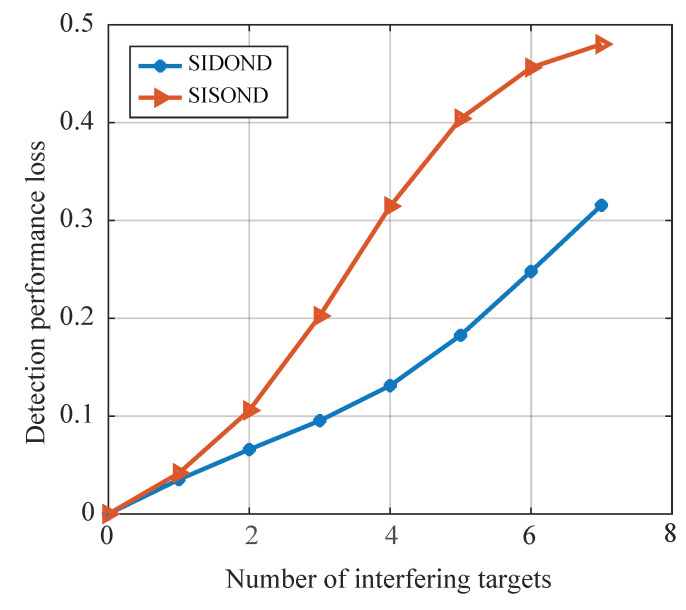
Average performance loss for SIDOND and SISOND in different interfering target environments.

Additionally, the average performance loss is defined as the average difference of all $P_{d}$ between multiple targets and homogeneous background within the range of [−15, 5] dB. Figure 11 displays the average performance loss of SIDOND and SISOND up to seven interferences, and the advantages of SIDOND become more apparent as the number of interfering targets increases.

**Figure 12:**
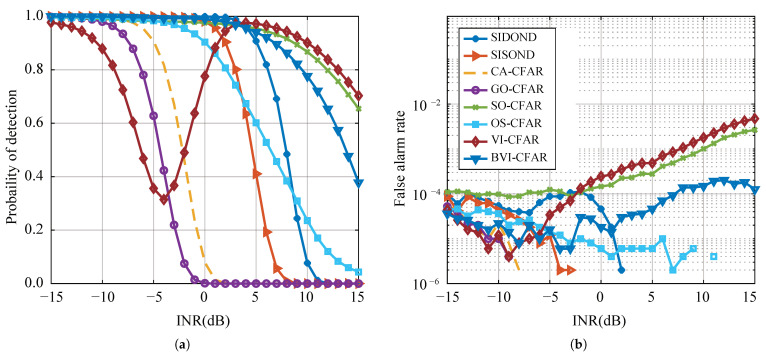
Detection performance (**a**) and false-alarm rate (**b**) with −2 dB target and one interference.

Furthermore, the impact of interference power on algorithm performance is analyzed to comprehensively evaluate the algorithm’s robustness. In this experiment, the target SNR is set to −2 dB, and the interference INR is set from −15 dB to 15 dB. Figure 12 shows that VI-CFAR, SO-CFAR, and BVI-CFAR sacrifice $P_{fa}$ to strengthen $P_{d}$, and the $P_{fa}$ of BVI-CFAR beyond the preset standard is the smallest among the three algorithms. SIDOND and OS-CFAR have the best robustness, with OS-CFAR maintaining some degree of $P_{d}$ even in cases of high INR, while the performance of SIDOND has almost no loss when the INR is lower than 5 dB.

#### 4.2.3. Complex Environment

**Figure 13:**
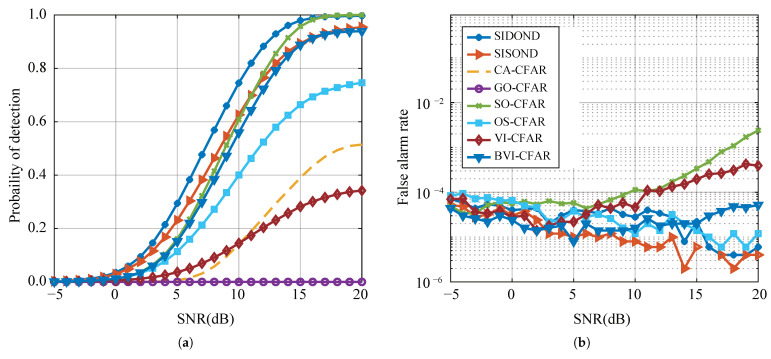
Detection performance (**a**) and false-alarm rate (**b**) in complex environments.

Due to the uneven distribution of clutter in the reference cell, clutter edges often cause false alarms. A set of target detection data is generated based on the principle that clutter edges are evenly distributed in reference cells and the CNR satisfies uniform distribution of $[10,20]\;\mathrm{dB}$. Up to four interfering targets appear in the reference cell with random amplitudes [1.0, 1.2] times that of the test target. The result is presented in Figure 13. Traditional methods exhibit a significant performance deterioration, especially SO-CFAR, due to the false alarm probability. SISOND’s performance deteriorates significantly when the target power is relatively high. On the contrary, SIDOND, with its dynamic-intelligent threshold mechanism, maintains high $P_{d}$ and low $P_{fa}$.

### 4.3. Visualization of the SIDOND

A visual explanation technique called gradient-weighted class activation mapping (Grad-CAM) is utilized to analyze the signal structure extracted by the SIDOND feature extractor. This method has enabled us to assess the effectiveness of the feature extraction process. Grad-CAM produces a rough localization map by utilizing the gradients of any target that enters the periodic activation function-based convolutional (PBCN) layer. The generated map highlights the critical areas in the image or signal. In other words, the weighted feature map in PBCN is obtained by back-propagating the gradient of the output category.

**Figure 14:**
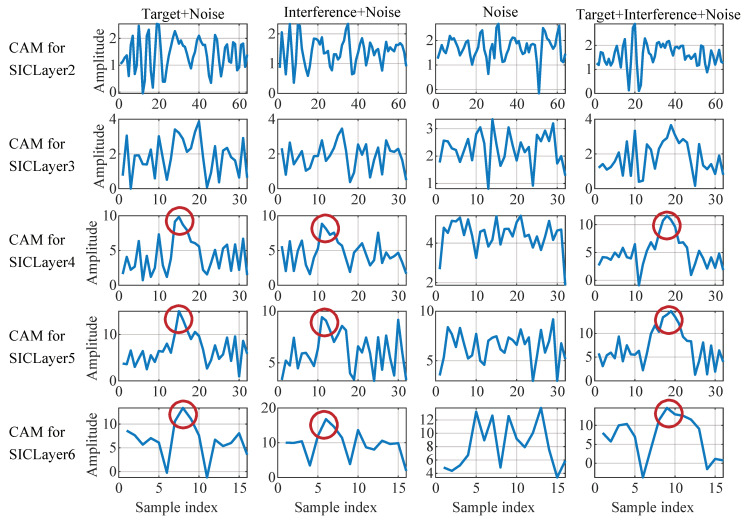
The attribution map of some layers. The four columns represent four scenarios of different input signals, and the five rows represent the activation mapping of five SICLayers in PBCN. The red circle represents the peak position and sinc shape extracted by network learning.

**Figure 15:**
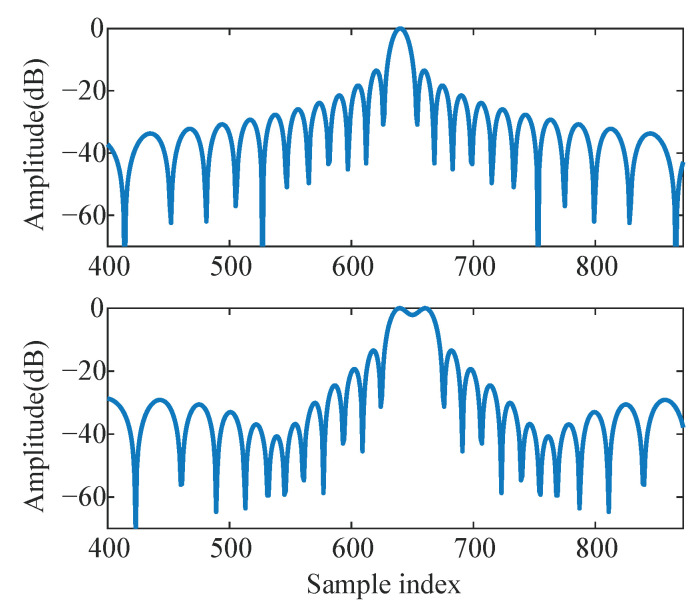
The magnitude of the echo from a target after matched filter (**top**) and the magnitude of the echo from a target and a interference after matched filter (**bottom**).

The features obtained by Grad-CAM are presented in Figure 14. In the case of an LFM signal, the target echo or the target and interference echo can be obtained from the matched filter output, as shown in Figure 15, without considering the noise. Figure 14 illustrates the features in different SICLayers corresponding to the echo signals visualized by Grad-CAM in four different scenarios, namely Target + Noise, Interference + Noise, Noise, and Target + Interference + Noise. It can be found that for the Target + Noise and Interference + Noise scenarios (the first two columns) that the deeper the PBCN layers, the more they resemble the sampling of the LFM signal, although with different peak positions. In the figure, the peak position and sinc shape are marked by the red circle. The PBCN fails to extract echo waveform-related information in the presence of noise. When dealing with the scenario Target + Interference + Noise, the SICLayer appears to sample the aliasing sinc function of the target and interference echo, as illustrated in Figure 15. This implies that the proposed PBCN progressively captures the representation of the target echo.

### 4.4. Computational Complexity Analysis

The computational complexity of an algorithm is a vital metric to gauge its performance [31]. The preprocessing of traditional methods involves matched filtering, whereas SIDOND and SISOND data require normalization. The training of SIDOND involves 30 epochs using an NVIDIA Quadro P4000 GPU with 8GB of memory, which takes around 10 h. The average runtime of a single detection is presented in Table 5. The mean-level CFAR, OS-CFAR, and VI-CFAR exhibit the lowest computational complexity. However, the computational complexity of the BVI-CFAR is comparable to that of the proposed SIDOND. Furthermore, the computational efficiency of SISOND can be enhanced through parallel computing on the GPU. In practical applications, pruning techniques can be utilized to improve computational efficiency [32].

## 5. Conclusions

This paper proposes a novel DNN-based approach to address the problem of target detection in complex scenarios. The proposed method utilizes a single-input dual-output network architecture consisting of a convolutional neural network with a periodic activation function for feature extraction from waveform intrinsic structure information. Additionally, two fully connected networks are employed to estimate the sufficient statistics and threshold impact factor, leading to a dynamic-intelligent threshold detection mechanism. The simulation results validate the efficiency and robustness of the proposed approach in challenging scenarios such as multiple targets, clutter edges, and their superposition. Furthermore, the visualization technique is adopted to demonstrate the effectiveness of the proposed network architecture.

## Figures and Tables

**Table 1:** Sensor simulation parameters.

Symbol	Significance	Value
*B*	Signal bandwidth	5 MHz
τ	Pulse Width	12.8 μs
$F_{s}$	Sampling frequency	5 MHz
*L*	Number of pulse sampling points	64
*v*	Target speed	$[-3400,3400]$ m/s

**Table 2:** The parameters of PBCN with different layers and nodes.

Label	The Parameters of *i*-th SICLayer							
	1	2	3	4	5	6	7	8
#1	(8,3,1,3)	(64,3,2,1)	(256,3,2,1)	–	–	–	–	–
#2	(8,3,1,3)	(64,3,2,1)	(128,3,1,1)	(256,3,2,1)	–	–	–	–
#3	(8,3,1,3)	(64,3,1,1)	(64,3,2,1)	(128,3,1,1)	(256,3,2,1)	–	–	–
#4	(8,3,1,3)	(64,3,1,1)	(64,3,2,1)	(128,3,1,1)	(256,3,1,1)	(256,3,2,1)	–	–
#5	(8,3,1,3)	(32,3,1,1)	(64,3,1,1)	(64,3,2,1)	(128,3,1,1)	(128,3,1,1)	(256,3,2,1)	–
#6	(8,3,1,3)	(16,3,1,1)	(32,3,1,1)	(32,3,2,1)	(64,3,1,1)	(128,3,1,1)	(256,3,1,1)	(256,3,2,1)

**Table 3:** TIF classification standards.

TIF	0	1	2	3	4	5	6	7	8	9	10
SNR/INR(dB)	(−*∞*,−13]	[−12,10]	[−9,8]	[−7,−6]	[−5,−4]	[−3,−3]	[−2,−1]	[0,0]	[1,2]	[3,4]	[5,*∞*)

**Table 4:** Comparison of performance with different signal model parameters.

Signal Bandwidth	Sampling Frequency	Pulse Width	Number of Pulse Sampling Points	Accuracy
5 MHz	5 MHz	12.8 us	64	0.972
4 MHz	4 MHz	16 us	64	0.971
4 MHz	4 MHz	32 us	128	0.986

**Table 5:** Runtime comparison for the processing of each detector.

Algorithms	Runtime (CPU)	Runtime (GPU)
SIDOND (proposed)	0.53 ms	0.0093 ms
SISOND	0.44 ms	0.0090 ms
Mean-Level-CFAR [3,4,5]	0.00013 ms	–
OS-CFAR [2]	0.00034 ms	–
VI-CFAR [9]	0.00052 ms	–
BVI-CFAR [11]	0.23 ms	–

**Figure A1:**
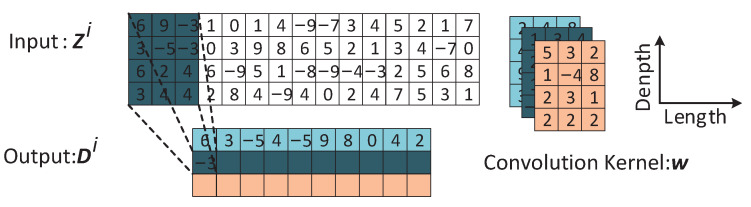
Convolution calculation process.

## Data Availability

The data presented in this study are available on request from the corresponding author. The data are not publicly available due to policy reasons.

## Appendix A. Initialization Scheme and Proof of Distribution

Assuming that the input data to the SICLayer is represented as $Z^{i}$, it has two dimensions: the depth dimension $L^{i}$ and the length dimension $H^{i}$. The output matrix of the convolutional layer is represented as $D^{i}$, and its dimensions are $L^{o}$ and $H^{o}$. Similarly, the output of the SICLayer is represented as $Z^{o}$, and its dimensions are $L^{o}$ and $H^{o}$. The convolution kernel is represented as w, which is a weight matrix with three dimensions: input depth $L^{i}$, kernel length $N_{k}$, and output depth $L^{o}$. Each convolution operation can be regarded as a vector dot product operation. Throughout the derivation process, the matrices are expanded into one-dimensional vectors. This does not affect the convolution result, but it simplifies the derivation process.

During the convolution process depicted in Figure A1, multiplication and summation operations are required. In order to analyze the data distribution conveniently, only the data from one convolution operation are considered at a time, which corresponds to the colored part of Figure A1. The length of each expanded vector is always $N_{k}L^{i}$. When considering a particular convolution operation, the data from $Z^{i}$, $Z^{o}$, $D^{i}$, and w are vectorized as $z^{i}$, $z^{o}$, $d^{i}$, and $\vec{w}$, respectively.

The data, after batch-normalization, meet the standard normal distribution. Then, input of the first SICLayer is $z^{i}∼N\left(0,1\right)$. The output of the convolutional layer is

(A1) $$d_{j}^{i}=\sum z^{i}⊙\vec{w}=z^{i}{\vec{w}}^{T}.$$

In the following derivation, the specific subscripts of the vectors are not considered since the Hadamard product can replace all the steps of the convolution operation. As shown in Figure 3, before calculating the periodic activation function, the data must be magnified by $N_{w}$. The initialization of $N_{w}$ in (11) and its value at this position are ignored, or it is set to 1 for simplicity.

The input of the first periodic activation function is $d^{i}$ and its variance $Var [d^{i}]=Var [z^{i}{\vec{w}}^{T}]=Var [{\vec{w}}^{T}]Var [z^{i}]$ [33]. When $w∼U\left(-c,c\right)$, the $Var [\vec{w}]=c^{2}/3$. In the first layer, $c=\sqrt{3/\left(N_{k}L^{i}\right)}$ and the Central Limit Theorem with weak Lindenberg’s condition [34,35] can be used to obtain $Var [d^{i}]=\left(N_{k}L^{i}\right)\left(c^{2}/3\right)=1$. Then $d^{i}∼N\left(0,1\right)$.

The output of the periodic activation function, $Z^{o}$, conforms to the arcsine distribution, which can be proven by showing that $D^{i}∼N\left(0,1\right)$ and $Z^{o}=\mathrm{Sin}\left(D^{i}\right)$. Specifically, it needs to be demonstrated that when $X∼N\left(0,1\right)$, $Y=\mathrm{Sin}\left(X\right)$ satisfies $Y∼\mathrm{ArcSin}\left(-1,1\right)$.

The cumulative distribution function of *X* is [36]

(A2) $$F_{X}\left(x\right)=P\left(X\leq x\right)=\frac{1}{2}+\frac{1}{2}erf\left(x/sqrt(2)\right)\approx \frac{1}{2}+\frac{1}{2}\tanh\left(\beta x\right),$$

where The value of β is 0.690. The probability quality of *X* lies on the interval [−3, 3] with 99.7% probability. Therefore, the cumulative distribution function of *Y* can be approximated as follows:

(A3) $$F_{Y}\left(y\right)=P\left(\sin\left(x\right)\leq y\right)=P\left(x\leq \arcsin\left(y\right)\right)\approx F_{X}\left(3\right)-F_{X}\left(-\arcsin\left(y\right)\right).$$

Putting (A2) into (A3), the $F_{Y}\left(y\right)$ can be formulated as

(A4) $$F_{Y}\left(y\right)=\frac{1}{2}\tanh\left(3\beta \right)-\frac{1}{2}\tanh\left(-\arcsin\left(y\right)\beta \right).$$

Using the Taylor expansion in $\arcsin\left(y\right)=0$, the $F_{Y}\left(y\right)$ is rewritten to

(A5) $$\begin{array}{c} F_{Y}\left(y\right)=\frac{1}{2}\tanh\left(3\beta \right)+\frac{\beta }{2}\arcsin\left(y\right)\approx \frac{1}{2}+\frac{1}{\pi }\arcsin\left(y\right). \end{array}$$

Then, $Y∼\mathrm{Arcsin}\left(-1,1\right)$. Simultaneously, $Z^{o}∼\mathrm{Arcsin}\left(-1,1\right)$. Next, the input $D^{i}$ before the non-first layer periodic activation function conforms to the standard normal distribution is only need to be proved. For the non-first floor, $Z^{i}∼\mathrm{Arcsin}\left(-1,1\right)$ and $c=\sqrt{6/\left(N_{k}L^{i}\right)}$. Using the Central Limit Theorem with weak Lindenberg’s condition, the variance of $d^{i}$ is

(A6) $$Var [d^{i}]=\left(N_{k}L^{i}\right)Var [{\vec{w}}^{T}]Var [z^{i}]=\left(N_{k}L^{i}\right)\left(c^{2}/3\right)\left(1/2\right)=1.$$

The random variable $D^{i}$ is normally distributed with mean 0 and variance 1. The initialization scheme used In this paper leads to the approximate normal distribution of data before the activation function, and the approximate arcsine distribution of data after the activation function.
